# Negative experiences and coping strategies to stressful situations by undergraduate University students during Covid- 19 lockdown period in Uganda

**DOI:** 10.4314/ahs.v23i4.16

**Published:** 2023-12

**Authors:** Ruth Ketty Kisuza, Saviour Kicaber, Ronald Olum, Dianah Rhoda Nassozi, Abel Wembabazi, Jennifer Namagembe, Enid Akot, Derrick Bary Abila, Felix Bongomin, Christopher Garimoi Orach

**Affiliations:** 1 School of Biomedical Sciences, College of Health Sciences, Makerere University Kampala Uganda; 2 School of Medicine, College of Health Sciences, Makerere University Kampala, Uganda; 3 School of Health Sciences, College of Health Sciences, Makerere University Kampala, Uganda; 4 Department of Medical Microbiology and Immunology, Faculty of Medicine, Gulu University, Gulu, Uganda; 5 Non-communicable and Infectious Disease Research (NIDER) Platform, Kampala, Uganda; 6 School of Public Health, College of Health Sciences, Makerere University Kampala, Uganda

**Keywords:** Stress, Mental health, coping strategies, negative experiences, and COVID-19

## Abstract

**Aim:**

To describe the negative experiences, coping strategies for stressful situations, and factors associated with COVID-19 worry among undergraduate students at Makerere University during the second COVID-19 lockdown in Uganda.

**Methods:**

Descriptive cross-sectional study at Makerere University. Negative experiences and coping strategies were measured using a semi-structured questionnaire formulated based on literature and findings from previous studies on mental health and COVID-19. Descriptive statistics and measures of association were done using Stata 13.

**Results:**

A total of 301 participants were enrolled. Their median age were 23 years. The majority were male (192, 64.0 %), single (269, 89.7 %,) and on normal academic progress (241, 80.3 %). 48.0 % of the participants were worried about COVID-19. Disruption of students' academics (277, 92.0 %) and financial problems (184, 61.1 %) were the most reported negative experiences. Coping strategies included listening to music (203, 67.4 %), talking to family and friends (191, 63.5 %,) and watching movies (174, 57.8 %). Female students than males (aOR: 2.0, 95% CI: 1.0–45.0, p= 0.118) and students without paid employment than those with paid work (aOR: 2.2, 95% CI: 1.0–5.0, p=0.109) were more worried about COVID-19.

**Conclusion:**

Nearly half of the students were worried about COVID-19, which disrupted their social and academic lives. The students utilized a myriad of coping strategies.

## Introduction

The coronavirus disease 2019 (COVID-19) first reported in December 2019 in Wuhan, China subsequently became a global pandemic in March 2020, causing severe disruptions and deaths to many people globally 1,2. With over 395 million cases and 5.7 million deaths worldwide, many countries are still grappling with its adverse effects on the economic, health, and education sectors, among others 3. These effects resulted mainly from the public health measures implemented to contain the spread of the virus. For example, most countries instituted nationwide lockdowns and travel restrictions 4-6.

The effects of the COVID-19 pandemic on the education sector cannot be overemphasized 7. For nearly two years, most learners in Uganda could not return to school due to the COVID-19-related shutdown of schools^8^, which equally had far-reaching effects on the mental health of the learners 9. The second COVID-19 lockdown was instituted in June 2021, when schools and universities had just been partially reopened to continue learning, especially for final-year students^10^. This was due to the surge in COVID-19 infections and related deaths in the country^11^.

A substantial amount of literature has been published on the effect of COVID-19 lockdowns on the mental health of different populations 12-17. For university students, the closing of universities led to an abrupt loss of personal contact with peers and faculty and the postponement of curricula, research, practical work, and exchange programs 18. The abrupt and often unprepared switch to online learning additionally evoked stress 19. Moreover, the loss of temporary jobs also compounded financial uncertainties 20.

Several studies have established that most university students worldwide had high levels of mental distress during the COVID-19 lockdown period 21-25. A study on the psychological impact of COVID-19 and lockdown among university students in Malaysia reported that the majority of the students (20.4%) experienced minimal to moderate levels of anxiety. The main stressors for students included financial constraints, remote online teaching, and uncertainties concerning academics, performance, and future career prospects 26. A study on depression, anxiety, and stress among Ugandan university students during the 2020 COVID-19 by Najjuka et al. found that the prevalence of mental health symptoms among participants was 80.7%, 98.4%, and 77.9% for depression, high levels of anxiety, and stress, respectively 27.

An important psychological factor affecting stressful life events such as COVID-19 on individuals' mental health is how individuals cope with stress 28. Coping is defined as the cognitive and behavioural efforts individuals employ to manage stress. Numerous coping styles have been identified. These include self-distraction, active coping, denial, substance use, use of emotional support, service of informational support, and behavioural changes 29. The negative impact of the COVID-19 pandemic and lock-downs on the mental health of university students is well documented 27,30,31. However, there is limited information on the different coping strategies used by university students when faced with other stressors associated with the COVID-19 lockdown. A study that assessed the mental health and various coping strategies among the general population living under imposed COVID-19 lockdown across the world showed that watching television, social networking, listening to music, sleeping, doing house chores, and eating well ranked among the most utilized coping strategies by all participants 32.

This study, therefore, set out to describe the negative experiences, related coping strategies to stressful situations, and factors associated with COVID-19 worry among undergraduate students at Makerere University during the second COVID-19 lockdown period of 2021 in Uganda.

## Methods

### Study design

We conducted a descriptive cross-sectional survey between September and December 2021.

### Study area

The study was conducted at Makerere University, Kampala, Uganda. Makerere University is the largest and third oldest institution of higher learning in Uganda. Makerere University has 9 colleges and one school. These include College of Health Sciences, College of Agricultural and Environmental Sciences, College of Business and Management Sciences, College of Computing and Information Sciences, College of Education and External Studies, College of Engineering Design, Art and Technology, College of Humanities and Social Sciences, College of Natural Sciences, College of Veterinary Medicine, Animal Resource and Biosecurity and the School of Law. Makerere University had a total population of about 38,000 students as of the 2020/2021 academic year. At the time of the study, only final-year students and those undertaking health professional programs were allowed to have physical learning on campus. In contrast, the rest studied online using Makerere University E-Learning Platform (MUELE) and video conferencing applications.

### Target population

All undergraduate students at Makerere University, 18 years older and in any year of study (first- fifth year) for the academic year 2020/2021 were targeted. An undergraduate student in this study was defined as one who was enrolled in any of the bachelor's degree programs offered at Makerere University at the time in which the study was conducted.

### Sample size

The sample size was calculated using Kish-Leslie formula.


$$N=\frac{Z^{2}p(1-p)}{d^{2}}$$

Where N = Sample size, Z = Standard deviation at 95% Confidence Interval (1.960), p = Expected level of stress and anxiety among university students, d = Precision of the estimate (5%).

The estimated levels of stress as a major outcome of the negative experiences resulting from the COVID 19 lock-down was taken as 54.7% a from previous cross-sectional study on stress and its sources among health professional students at Makerere University, Uganda by Amanya et al^33^. Therefore, the sample size for this study n = 372.

### Sampling procedure

Due to the strict COVID 19 measures that were implemented by the Government of Uganda to curb the spread of COVID 19, we used convenience sampling in which all students who were able to access the link to the online questionnaire were involved in the study. We identified different student WhatsApp groups with the help of class representatives and research assistants were we frequently shared the study link and invited the students to participate in the study.

### Data collection

Data were collected using a semi-structured comprehensive questionnaire formulated based on literature and findings from previous studies on mental health and COVID-19^28^,^34^,^35^. The questionnaire was composed of different sections. The first section included social demographic characteristics like age, sex, marital status, college of study, religion, duration of bachelor's program, year of study, form of sponsorship, sources of financial support, employment status, pending retake and history of mental health diagnosis. The second section of the questionnaire investigated COVID-19 related variables. This included source of information on COVID-19 information, a Likert scale measuring the levels of worry about COVID-19, ever testing for COVID-19, and having a friend, relative or neighbour that succumbed to COVID-19. The third part of the questionnaire explored the negative experiences of students during the COVID-19 lockdown. This section constituted of a list of possible negative experiences developed by the study investigators that could have resulted from the second COVID-19 lockdown and students were free to choose from any of the negative experience that applied to them during the lockdown. Lastly, the fourth and last part of the questionnaire was about coping strategies to stressful situations by students. Like the third part of the questionnaire, a list of both positive and negative coping strategies was listed in this part of the questionnaire from which students could choose the different coping strategies that they used.

### Data management and statistical analysis

Data were collected using Google forms and completed questionnaires/data were downloaded and exported to Microsoft Excel for cleaning and coding. Statistical analysis was done using STATA 13 (StataCorp LLC, College Station, Texas, USA). Descriptive statistics were used to summarize socio-demographic variables. All categorical variables were summarized as frequencies and proportions whereas continuous variables as mean (standard deviation) or median (interquartile range, IQR) for parametric and non-parametric conditions, respectively. We used bi-variable and multi-variable logistic regression to determine the association between demographic characteristics and COVID-19 worry. COVID-19 worry which was measured using a five-point likert scale (not at all worried, not very worried, somewhat worried, very worried and extremely worried) was recoded to dichotomous variable with “not at all worried” as “Not worried” and the other options as “Worried.”. For multivariable logistic regression, we included independent variables with a P-value less than 0.2 as well as independent variables that have shown to be associated with COVID-worry or we thought could be associated with COVID-19 worry. We reported Crude odds ratios, adjusted odds ratios (aOR), P-values and 95% confidence intervals. A p<0.05 was considered statistically significant.

### Ethical considerations

This study was approved by Mulago Hospital Research and Ethics committee under the reference number MHREC-2021-37. Participation in the study was entirely voluntary and all participants provided consent before completing the questionnaire.

## Results

### Demographic characteristics of participants

A total of 301 (response rate = 81%) participants were included in this study. The median age of the participants was 23 (IQR, 20-30) years. Students from all classes and colleges at Makerere University were included in our study. Majority of the participants were male (192, 64.0%,), single (269,89.7%,) and were on normal academic progress (241,80.3%,) before the second COVID 19 lockdown. Table 1 summarizes the demographic characteristics of the participants.

**Table 1:** Socio-demographic characteristics of the study participants

Variable	Frequency	Percentage (%)
**Sex**		
Male	192	64.0
Female	108	36.0
**College**		
College of Agricultural and Environmental sciences.	28	9.3
College of Business and Management Sciences.	21	7.0
College of Computing and Information Sciences.	8	2.7
College of Education and External Studies	31	10.3
College of Engineering, Design, Art, and Technology	33	11.0
College of Health Sciences	83	27.7
College of Humanities and Social Sciences	24	8.0
College of Natural Sciences	15	5.0
College of Veterinary Medicine, Animal Resource and Biosecurity	32	10.7
School of Law	25	8.3
**Marital status**		
Single	269	89.7
Married	9	3.0
Prefer not to say	22	7.3
**Religion**		
Muslim	20	6.7
Roman Catholic	83	27.7
Protestant	108	36.0
Born Again	68	22.7
Seventh Day Adventist (SDA)	7	2.3
Others	14	7.6
**Financial support**		
Parents/ relatives	220	73.3
Self	63	21.0
Friends	3	1.0
Other	14	4.7
**Type of sponsorship**		
Government Sponsorship	119	41.0
Private Sponsorship (Parents and Guardians)	136	45.3
Private Sponsorship (Self sponsored)	30	10.0
Non-Government Organization	15	5.0
**Program duration**		
3 years	123	41.0
4 years	97	32.0
5 years	80	26.7
**Year of study**		
Year 1	40	13.3
Year 2	72	24.0
Year 3	117	39.0
Year 4	53	17.7
Year 5	18	6.0
**Pending retake**		
Yes	59	19.7
No	241	80.3
**Had Paid employment**		
Yes	45	15.2
No	252	84.9
**Diagnosed/History of mental Health diagnosis**		
Yes	61	20.3
No	239	79.7

### COVID-19 Information

Table 2 summarizes the COVID-19-related information among the study participants. Overall, close to half the students were worried about COVID-19 (144, 48.0%,), had never tested for COVID-19 (209,69.7%,) and had a friend, relative or neighbour who succumbed to it (163,54.3%,).

**Table 2:** COVID-19 information of the study participants

Variable	Frequency	Percentage (%)
**Ever tested for COVID 19**		
Yes	91	30.3
No	209	69.7
**Was positive for COVID 19**		
Yes	26	28.6
No	65	71.4
**Had a friend/relative/neighbour who tested positive for COVID 19**		
Yes	232	77.6
No	67	22.4
**Lost friend/ relative/ neighbour to COVID 19**		
Yes	163	54.3
No	137	45.7
**Source of information about COVID 19**		
Television/radio	96	32.0
Social media	122	40.7
Ministry of health online platforms	53	17.7
WHO/CDC platform	7.67	7.7
Neighbours /peers/ relatives	2.0	2.0
**Worried about COVID 19**		
Not at all worried	36	12.0
Not very worried	41	13.7
Somewhat worried	79	26.3
Very worried	59	*19.7
Extremely worried	85	28.3

### Negative experiences of students during the second lockdown in Uganda

The negative experiences reported during the lockdown varied across respondents. However, disruption of student academics (277, 92.0%), financial problems (184,61.1%,), lack of close and intimate friends (129,42.9%,) and fear of COVID 19 (126, 41.9%,) were the most reported negative experiences. Table 3 shows the negative experiences of students during the second COVID 19 lockdown in Uganda.

**Table 3:** Negative experiences of students during the second lockdown in Uganda

Negative experience	Frequency	Percentage (%)
Disruption of my academics	277	92.0
Financial problems	184	61.1
Lack of close and intimate friends	129	42.9
Fear of COVID-19	126	41.9
New responsibilities of life	107	35.5
Parent expectations	102	33.9
Changes in sleeping pattern	79	26.2
Change in eating pattern	65	21.6
Death of significant one	53	17.6
Conflicts with close one	51	16.9
Decline in personal health	51	16.9
Homesickness in hostel	21	7.0
Change in religious beliefs	18	6.0
Engagement/ marriage	13	4.3

### Coping strategies to stressful situations by students during the second lockdown in Uganda

Most students used positive coping strategies to deal with the negative experiences that resulted from the COVID 19 lockdown. Listening to music (203, 67.4%,), talking to family and friends (191, 63.5%,), watching movies (174, 57.8%,) and physical exercise (140,46.5%,) were the most reported coping strategies to stressful situations. However, some students reported ideating about suicide (7, 2.3%,) and drinking alcohol (24, 7.9%,) as their coping strategies (Table 4).

**Table 4:** Coping strategies to stressful situations by students during the second lockdown

Coping strategy	Frequency	Percentage (%)
Talk to family and friends	191	63.5
Physical exercise	140	46.5
Watching movies / television	174	57.8
Listening to music	203	67.4
Drink Alcohol	24	7.9
Talking to a professional (counseling)	39	13.0
Take a walk	113	37.5
Meditation	92	30.6
Lock myself in my room and cry	63	20.9
Thinking about Suicide	7	2.3
Others	28	9.3

### Factors associated with COVID-19 worry during the second COVID 19 lockdown

From multi-variable logistic regression, female students (AOR: 2.0, 95% CI: 1.0 — 5.0, p=0.118) were more worried about COVID-19 than males. Additionally, students without paid employment (AOR: 2.2, 95% CI: 1.0 — 5.0, p=0.109) were more worried about COVID-19 than students with paid employment, however this was not statistically significant. Detailed results for both bi-variable and multivariable analysis are shown in Table 5.

**Table 5:** Factors associated with COVID-19 worry during the second COVID 19 lockdown

Factor	COR (95% CI)	P-Value	AOR (95% CI)	P-value
**Sex**				
Male	1 (reference)		1 (reference)	
Female	2.04 (0.89 -4.68)	0.089	2.04 (0.835-4.979)	0.118
**Religion**				
Muslim	1 (reference)		1 (reference)	
Roman Catholic	0.66 (0.08-5.87)	0.715	0.59 (0.064-5.47)	0.065
Protestant	0.65 (0.08-5.51)	0.694	0.52 (0.059-4.64)	0.560
Born Again	0.19 (0.25-1.62)	0.131	0.18 (0.02-1.55)	0.120
SDA	0.13 (0.01-1.76)	0.126	0.13 (0.01-1.82)	0.128
Others	0.13 (0.01-1.34)	0.087	0.09 (0.01-1.06)	0.058
**Marital status**				
Single	1 (reference)		-	-
Married	0.91 (0.11-7.73)	0.938	-	-
Prefer not to say	0.83 (0.23-2.97)	0.777	-	-
**College of study**				
College of Agricultural and Environmental sciences	1 (reference)		-	-
College of Business and Management Sciences	1.14 (0.17-7.52)	0.892	-	-
College of Computing and Information Sciences.	0.36 (0.05-2.66)	0.316	-	-
College of Education and External Studies	1.08 (0.19-5.85)	0.929	-	-
College of Engineering, Design, Art and Technology	0.87 (0.18-4.27)	0.864	-	-
College of Health Sciences	0.63 (0.17-2.39)	0.495	-	-
College of Humanities and Social Sciences	1		-	-
College of Natural Sciences	0.78 (0.12-5.27)	0.799	-	-
College of Veterinary Medicine, Animal Resource and Bio-Security	0.65 (0.14-2.99)	0.579	-	-
School of Law	2.88 (0.28-29.64)	0.374	-	-
**Course duration**				
3 years	1 (reference)		1 (reference)	
4 years	0.85 (0.358-2.02)	0.714	0.92 (0.36-2.39)	0.869
5 years	0.62 (0.27-1.47)	0.280	0.65 (0.25-1.66)	0.364
**Year of study**				
Year 1	1 (reference)		-	-
Year 2	1.39 (0.45-4.34)	0.571	-	-
Year 3	1.38 (0.49-3.93)	0.541	-	-
Year 4	1.38 (0.41-4.66)	0.601	-	-
Year 5	1.41 (0.26-7.78)	0.692	-	-
**Pending retake**				
Yes	1 (reference)			
No	1.23 (0.53-2.86)	0.637	-	-
**Diagnosed/History of mental Health diagnosis**				
Yes	1 (reference)		**-**	**-**
No	0.98 (0.975-0.41)	0.975	**-**	**-**
**Financial support**				
Parents/ relatives	1 (reference)		**-**	**-**
Myself	0.67 (0.29-1.56)	0.358	**-**	**-**
Friends	0.05 (0.01-0.65)	0.021	**-**	**-**
Other	0.68 (0.14-3.22)	0.624		
**Type of sponsorship**				
Government Sponsorship	1 (reference)		1 (reference)	
Private Sponsorship (Parents and Guardians)	1.16 (0.53-2.55)	0.706	0.92 (0.39-2.22)	0.866
Private Sponsorship (Self sponsored)	0.516 (0.18-1.49)	0.220	0.58 (0.19-1.79)	0.346
Non-Government Organization	1.88 (0.23-15.45)	0.555	1.65 (0.19-14.57)	0.650
**Had Paid employment**				
Yes	1 (reference)		1 (reference)	
No	2.17 (0.94-5.01)	0.069	2.17 (0.84-5.61)	0.109

## Discussion

Overall, about half of the students were apprehensive about COVID-19 and contracting the disease. This could be because Uganda's second COVID 19 lockdown period was characterized by many COVID-19 related deaths 36. Moreover, most students reported losing at least a friend, neighbour, or a relative to COVID-19. These findings are consistent with similar studies on assessing levels of fear of COVID-19 among university students 30,37.

The study also found that most students had never tested for COVID-19. Important to note is that the second COVID-19 lockdown period was characterized by widespread cough and flu like symptoms among Ugandans associated with the Delta Variant of COVID-19^11^. Therefore, we anticipated that majority of the students should have tested for COVID-19, which was not the case. A possible explanation for this is the fear of testing positive for COVID-19 and consequently having to deal with its associated stress and anxiety 38. This could also be explained by the fact that the COVID-19 test was expensive, making it hard for students to afford.

On the negative experiences of students during the second COVID-19 lockdown in Uganda, the most obvious finding to emerge from the study is the disruption of studies/academics as the most reported negative experience. This is because most students had just resumed their studies following partial reopening of the country which allowed for continuation of learning for university students.

The study also showed that financial problems and lack of close and intimate friends were among the frequent negative experiences of Makerere University students. These results are in agreement with a study on the impact of lockdown stress and loneliness among students in Germany during the COVID-19 pandemic on mental health among university students which reported that most students (30.56%) felt lonely 39. Additionally, our findings agree with similar studies on COVID-19 related mental health burdens among low-income earners of western and South western Uganda which found that anger and anxiety increased /span>among low-income earners 40,41.

Very little was found in the literature on the different coping strategies used by students when faced with stressful situations and challenges that resulted from the COVID-19 lockdown. This study showed that most students at Makerere University used positive coping strategies to deal with stressful situations. Listening to music, talking to family and friends, watching movies and physical exercise were the most commonly used coping strategies 32. This could be because, following the second COVID-19 lockdown in 2021, the Ministry of Health in Uganda channelled a lot of effort and resources to educate Ugandans about mental health and coping mechanisms to stressful situations through online campaigns, TV and radio talk shows, and posters. However, some students reported ideating about suicide as their coping strategy. This troubling finding helps us understand the somewhat high prevalence of suicide among university students 42. Additionally, it underscores the need for university students to have more readily available counseling services.

Our study further revealed that socio-demographic characteristics like sex, paid employment was associated with worrying about COVID-19. Female students were more worried about COVID-19 than males 39. Additionally, students without paid work were more worried about COVID-19 than those with paid employment. However, these findings were not statistically significant.

### Study strengths

Our findings contribute in several ways to our understanding of the negative experiences, coping strategies to stressful situations and factors associated with COVID-19 worry by undergraduate students at Makerere University during the second COVID-19 lockdown in Uganda and provides a basis for further research.

### Study limitations

We used convenience sampling to collect our research data which missed the views of the students who were offline/unable to access the internet during the study period. Also, WhatsApp used as a platform for data collection makes our results prone to selection bias. The study questionnaire was self-administered and thus subject to reporting and recall bias. Also, our questionnaire was not statistically validated for its reliability and it did not use a standard tool to access for negative experiences and coping strategies. We also acknowledge that a question to measure the levels of perceived stress and anxiety of the students using a standard tool would have added power to the study. Furthermore, the sample size from different colleges at the University was not equal and stratified which might have skewed the results.

## Conclusion

About half of the students were very worried about COVID-19. The most reported negative experiences by students were disruption of academics, financial problems and lack of close and intimate friends. Majority of students engaged in positive coping strategies such as listening to music, talking friends/family and physical exercise, and watching movies to deal with stressful situations during the COVID-19 lockdown.

## Recommendation

The University administration and stakeholders should avail more mental health counselling and awareness services for students in future pandemics.

## Data sharing statement

The data used to support this study's findings will be uploaded and made publicly accessible on Mendeley data.
